# Manipulation of Ovarian Function Significantly Influenced Sarcopenia in Postreproductive-Age Mice

**DOI:** 10.1155/2016/4570842

**Published:** 2016-09-22

**Authors:** Rhett L. Peterson, Kate C. Parkinson, Jeffrey B. Mason

**Affiliations:** Department of Animal, Dairy and Veterinary Sciences, Center for Integrated BioSystems, School of Veterinary Medicine, Utah State University, Logan, UT, USA

## Abstract

Previously, transplantation of ovaries from young cycling mice into old postreproductive-age mice increased life span. We anticipated that the same factors that increased life span could also influence health span. Female CBA/J mice received new (60 d) ovaries at 12 and 17 months of age and were evaluated at 16 and 25 months of age, respectively. There were no significant differences in body weight among any age or treatment group. The percentage of fat mass was significantly increased at 13 and 16 months of age but was reduced by ovarian transplantation in 16-month-old mice. The percentages of lean body mass and total body water were significantly reduced in 13-month-old control mice but were restored in 16- and 25-month-old recipient mice by ovarian transplantation to the levels found in six-month-old control mice. In summary, we have shown that skeletal muscle mass, which is negatively influenced by aging, can be positively influenced or restored by reestablishment of active ovarian function in aged female mice. These findings provide strong incentive for further investigation of the positive influence of young ovaries on restoration of health in postreproductive females.

## 1. Introduction

In many species, dietary restriction is proven to be beneficial to health and life span. In primitive species, removal of gonadal germ cells has a similar positive effect on health and life span. These observations suggest a relationship between reproduction and health, but this relationship has been difficult to define, particularly in mammals. In female mammals, dietary restriction increases health span but also induces ovarian germ cell quiescence.

A prominent example of the relationship between chronological life span, reproductive life span, and health is seen in human females. Chronological life span in humans has been extended dramatically over the last century. However, the timing of menopause or the end of the reproductive life span in human females has remained relatively constant over this same time period. Prior to reproductive decline, females hold a significant health advantage over males of the same age. However, at the time of reproductive decline, the increase of disease risks in women significantly outpaces those of men. This dependence on reproductive function for the maintenance of health is exemplified in women with premature ovarian failure, who suffer from a decline in health at a much younger age than in women with traditional menopausal timing.

In primitive species, the reproduction-related effects on health span are thought to be due to the preservation of young, gonadal somatic cells which are protected from active germ cell signaling by removal of the germ cells. Previously, we replaced senescent ovaries of postreproductive female mice with young ovaries. The result of this manipulation was an increase in life span similar to that observed with dietary restriction [1]. We also detected a decrease in cardiomyopathy and osteoarthritis at death in transplant recipients [2, 3]. In the current experiments, we tested skeletal muscle function, body composition, and glucose metabolism to determine how closely exposure of postreproductive female mice to young transplanted ovaries recapitulates the health span effects seen with dietary restriction. Exposure of postreproductive female mice to young transplanted ovaries significantly influenced body composition but not skeletal muscle function or glucose metabolism.

## 2. Material and Methods

### 2.1. Mice

The CBA/J strain (used in the current study) and the DBA strain of mice are unique in that they prematurely lose their ovarian follicles, becoming reproductively senescent by 10–12 months of age [4–6]. A reduction of ovarian follicles in the human is associated with the onset of menopause. For this reason, CBA/J mice may serve as an appropriate experimental model to study age-related changes in human reproduction [7, 8].

Twenty-one-day- and eight-month-old CBA/J strain female mice were obtained from Jackson Laboratory (Bar Harbor, ME). The 14-month-old female CBA/J mice were obtained from the National Institute on Aging rodent colony. All mice were housed in a standard laboratory animal environment (fresh filtered air, 15 changes/h; temperature, 21 ± 2°C; humidity, 50 ± 20%; and light-dark cycle, 12 : 12 h). The mice were housed individually in ventilated cages (Green Line IVC Sealsafe Plus, Tecniplast, West Chester, PA, USA) on corn cob bedding (7097 Corncob, Harlan Teklad, Bartonville, IL, USA) changed once a week, with added enrichment, in a specific-pathogen-free colony where pathology on sentinel mice was done quarterly and pathological results showed no breach in this status. The mice received deionized water and a certified laboratory diet* ad libitum* (2018 Teklad Global 18% Protein Rodent Diet, Harlan Teklad, Bartonville, IL, USA). Mice were maintained in an American Association for Accreditation of Laboratory Animal Care- (AAALAC-) approved facility in accordance with the National Institutes of Health animal-use guidelines. Animal care and use protocols were developed under National Research Council guidelines found in the Guide for the Care and Use of Laboratory Animals. This project was approved by the Utah State University Institutional Animal Care and Use Committee.

Anesthetics were used during surgery (see Surgical Procedures) and analgesia was provided for 48 hours postoperatively and longer if deemed necessary. Mice were euthanized by cervical dislocation. Immediately after cervical dislocation, thoracotomy was performed followed by rapid exsanguination via cardiocentesis. The heart and arterial tree were then removed from the mouse. Animals with acute but not severe weight loss were treated with subcutaneous fluids and moistened food. Animals with acute but not severe urine staining or rectal/vaginal prolapse were manually cleaned and treated with Desitin®. Mice were monitored at least twice daily and weights were recorded monthly, more frequently when concerns arose. Aged, moribund mice found with overt clinical signs (catatonia) were euthanized. Criteria for euthanasia specific for aged mice were determined in coordination with the attending veterinarian and included but were not limited to mice found in poor condition with or without crusting around the perineum and diarrhea, urine staining, persistent vaginal prolapse, chronic vulva/rectal swelling, kyphosis, respiratory distress, anorexia, poor coat condition and lack of grooming, moribund mentation, hind-limb weakness/paresis, wounds not healing, limited mobility, neoplastic growth, and unusual weight loss (or gain). Average weight loss in aged, female CBA/J mice from peak weight to death is approximately 12% per month [9]. An increased rate of weight loss but not total weight loss was the most critical factor for determining a moribund state. Unexpected deaths were uncommon but included neoplastic growths (most commonly mammary), decubitus ulcers (extremely old animals), and uncontrolled cataleptic seizures (normally between 11 and 13 months of age).

### 2.2. Experimental Design

**Figure 1:**
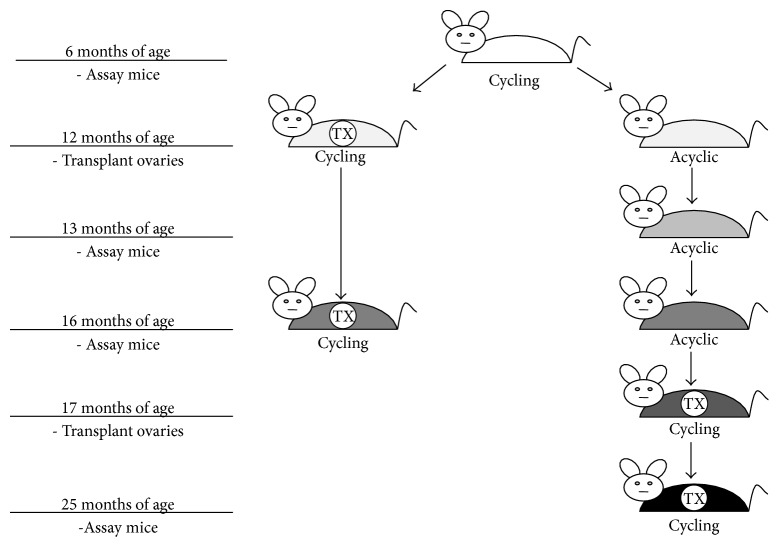
Experimental design: six-month-old control mice (*n* = 10), 13-month-old control mice (*n* = 5), 16-month-old control mice (*n* = 5), 16-month-old recipient mice (*n* = 5), and 25-month-old recipient mice (*n* = 5).

Animals were randomly assigned to control or ovarian transplantation groups as follows (Figure 1).

Controls consisted of the following.

 Six-month-old control mice: reproductively cycling mice were evaluated at six months of age (*n* = 10).
 Thirteen-month-old control mice: acyclic mice were evaluated at 13 months of age (*n* = 5).
 Sixteen-month-old control mice: acyclic mice were evaluated at 16 months of age (*n* = 5).

Ovarian transplants consisted of the following.

 Sixteen-month-old recipient mice: recipient animals remained intact but were acyclic at 12 months, at which time their senescent endogenous ovaries were removed and replaced with a pair of active donor ovaries from a 60-day-old mouse. These reproductively cycling mice were evaluated at 16 months of age (*n* = 5).
 Twenty-one-month-old recipient mice: recipient animals received at 14 months were acyclic. At 17 months, their senescent endogenous ovaries were removed and replaced with a pair of active donor ovaries from a 60-day-old mouse. These reproductively cycling mice were evaluated at 25 months of age (*n* = 5).

### 2.3. Age at Manipulation

Rodents do not undergo menopause but instead have an estropause-like decrease in reproductive function. Reproductive decline in CBA/J mice usually begins with irregular cycles at 8–10 months of age. At 11 months of age, many CBA/J mice have become reproductively incompetent [10] with a complete loss of oocytes by 12 months of age. Ovarian transplantation surgeries were conducted at 12 and 17 months of age. All 12- and 17-month-old mice used in these experiments displayed a complete lack of reproductive cycling prior to transplantation, as determined by vaginal cytology.

### 2.4. Surgical Procedures

Twelve-month- and 17-month-old animals underwent bilateral ovariectomy (OVX) and subsequent ovarian transplantation and received a pair of 60-day-old ovaries from a donor mouse of the same strain. Bilateral ovarian transplantation surgeries were performed as previously described [11]. Briefly, the ovaries were exposed by paralumbar incision under anesthesia (50–100 mg/kg ketamine, 10–15 mg/kg xylazine, and 2-3 mg/kg acepromazine, IP) and removed by incising the ovarian bursa opposite the ovarian hilum. The ovary was gently removed from the ovarian bursa and excised by clamping the ovarian hilum to prevent bleeding. Excised ovaries were placed in cold saline prior to transfer/replacement. After transfer/replacement, the ovarian bursa was closed with one to three sutures of 10-0 Ethilon monofilament (Ethicon, Inc.). The abdominal wall was sutured with 5-0 chromic gut (Ethicon, Inc.), and the skin was closed with 9 mm wound clips (MikRon Precision, Inc.).

Data on vaginal cytology were collected for at least 10 consecutive days pre- and postoperatively to ensure (1) cessation of cycling at 12 months of age and (2) success of the ovarian transplantation procedure. Daily vaginal cytology was reinitiated beginning 10–14 days postoperatively. One estrous cycle was defined as the period from the day nucleated epithelial cells first appeared (i.e., proestrus) to the day preceding the next appearance of nucleated epithelial cells in the vaginal smear, provided there was a period of leukocytic presence (i.e., diestrus) in between. Estrus was determined by the presence of large, squamous epithelial cells, with or without nuclei. No immunosuppressive techniques were employed and no evidence of graft-versus-host disease was detected after transplantation or at death. Each female was housed individually.

### 2.5. Exclusion Criteria

Mice that displayed cytological evidence of gonadal input prior to surgery at 12 or 17 months of age were excluded from analysis. Gonadal input was defined as cyclic changes on vaginal cytology, presumably due to cyclic influence of ovarian hormones. No gonadal input was defined as the lack of cyclic changes on vaginal cytology. Ovarian transplant recipients that failed to display evidence of gonadal input postoperatively based on vaginal cytology were also excluded from analysis. Gonadal input was determined by vaginal cytology analysis, as described in Surgical Procedures. Mice that displayed no cyclic activity on vaginal cytology for a 10-day period before and/or after surgery were determined to have no gonadal input for said period. Mice that displayed at least one full estrous cycle in a 10-day period before and/or after surgery were determined to have gonadal input for said period. Mice that fit these criteria were the only mice used for analysis throughout this study.

### 2.6. Indices of Health Span

Not all groups were subjected to each test of health span. Importantly, the 25-month-old group was often intolerant of metabolic or stressful manipulations and was included only in the magnetic resonance imaging (MRI) analysis procedures.

#### 2.6.1. Skeletal Muscle Function-Inverted Cling Grip Test

The inverted cling grip test (ICGT) is a measure of overall strength and muscular endurance of the mouse [12]. This test consisted of placing the mouse on a cage-like wire grid and then inverting the grid over a padded surface. The testing device was a custom designed 43 cm square of wire mesh consisting of 12 mm squares of 1 mm diameter wire, surrounded by a 4 cm deep wooden border to prevent mice from climbing to the other side of the screen. Each mouse was placed in the center of the inverted screen, parallel to the floor. Next, the screen was tilted up 90 degrees to cause the mouse to grab the screen tightly. The screen was subsequently turned another 90 degrees, again parallel with the floor, but flipped so that the mouse was positioned upside down hanging suspended from the wire grid. The latency before the mouse lost its grip from the wire grid and fell to the padded surface at the bottom of the device was recorded to a maximum of 2 hours. Because of the influence of circadian rhythms, the time of day for testing was kept constant.

#### 2.6.2. Metabolic Function-Glucose Tolerance Test

An intraperitoneal (IP) glucose tolerance test (GTT) was performed in mice that were feed-deprived for 4-5 hours. We chose a 4-5-hour fast due to the metabolic differences between humans and mice and additionally because our aged females did not respond well to an overnight fast [13]. Blood glucose levels were measured using FreeStyle Freedom Lite Blood Glucose Monitoring System (Abbott Diabetes Care Inc., Alameda, CA, USA) from blood droplets obtained from a small nick at the tip of the tail, 2 h prior to testing, again immediately prior to glucose administration (*t*_0_), and at 15, 30, 60, and 120 minutes after injection of 15% D-glucose (2.8 g/kg lean body mass). Calibration of the FreeStyle Freedom Lite Blood Glucose Monitoring System was performed using control test solutions provided by the manufacturer.

Two drops of blood were collected, the first of which is discarded. The second drop was applied directly to a FreeStyle Freedom Lite test strip to obtain a blood glucose reading expressed as mg/dL. Glucose measurements at each time point were plotted and results were expressed as the area under the curve (AUC) for the 120-minute assay. The AUC corresponding to each animal was calculated by the trapezoid method [14], using as reference each individual baseline blood glucose measurement prior to glucose administration (*t*_0_). The sum of the trapezoidal areas between the 0-, 15-, 30-, 60-, and 120-minute time points corresponding to each animal was computed to obtain the AUC. The relative area values are reported as a percentage of the mean AUC of the six-month-old control cohort, which was defined as 1.0.

#### 2.6.3. Body Composition-Magnetic Resonance Imaging

Alterations in mouse whole body composition were assessed using an EchoMRI-700 Magnetic Resonance Imaging system (EchoMRI, Houston, TX, USA). The EchoMRI-700 system is housed in a dedicated area to minimize stress and the entry of disease or contaminants. Prior to each run, the system was calibrated using a standard provided by Echo Medical System. Each mouse was weighed and placed into an appropriate size tube. The tube was then placed in the EchoMRI-700 machine for measurements (~1 min). The output information included lean tissue mass, fat mass, free water, and total body water. No anesthesia was required for this procedure.

### 2.7. Statistical Analysis

Statistical analysis was performed using GraphPad Prism 7.01 (GraphPad Software, Inc., La Jolla, CA). A D'Agostino-Pearson omnibus test was used to determine normality. Data were analyzed with two-factor ANOVA and a Tukey-Kramer* post hoc* test was used to determine difference between groups. Individual treatments were further analyzed by paired Student's *t*-test, two-tailed, unequal distribution of variance assumed. Test results were considered significant for *P* < 0.05.

## 3. Results

### 3.1. Skeletal Muscle Function-ICGT

**Figure 2:**
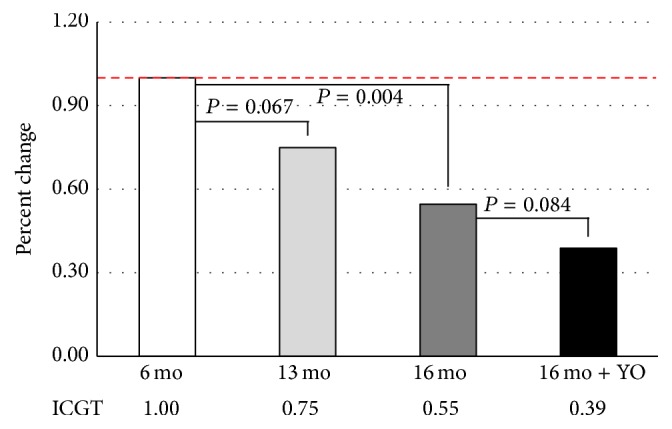
Inverted cling grip test. Grip strength decreased significantly with age. This decrease in grip strength was not significantly influenced by young ovaries (16 mo + YO). Values are reported as a percentage of the values in adult, six-month-old mice.

Performance on the ICGT defined as the average time (duration in seconds) before falling from the grid declined with age and with ovarian transplantation. The effect of age on overall muscle strength, as measured by the ICGT, is summarized in Figure 2. A significant age-related decline in grip strength was found in 16-month-old control mice (45% reduction, *n* = 5) and tended to be reduced in 13-month-old mice (25% reduction, *n* = 5), compared with six-month-old controls (*n* = 10). Sixteen-month-old recipient mice with young ovaries tended to fall sooner than the 16-month-old control mice (29% reduction, *P* = 0.085, *n* = 5).

### 3.2. Metabolic Function-GTT

**Figure 3:**
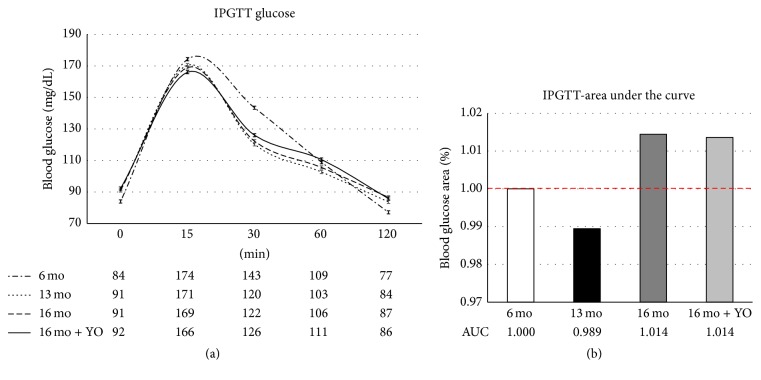
Metabolic function. The IPGTT assay detected no significant difference in glucose tolerance between six, 13-, and 16-month-old mice. Ovarian transplantation had no influence on this parameter. Values are reported as (a) blood glucose values for group glucose curves and for (b) the area under the curve, as a percentage of the values in six-month-old mice.

Because all groups did not have equivalent fasting glucose levels, glucose measurements at each time point were plotted and results were expressed as the area under the curve (AUC) for the 120-minute assay. AUC calculations for Figure 3 data show that, during IPGTT, both 16-month-old control (*n* = 5) and transplant recipient mice (*n* = 5) displayed no significant change in glucose tolerance, compared with young controls (*n* = 10).

### 3.3. Body Composition-MRI

**Figure 4:**
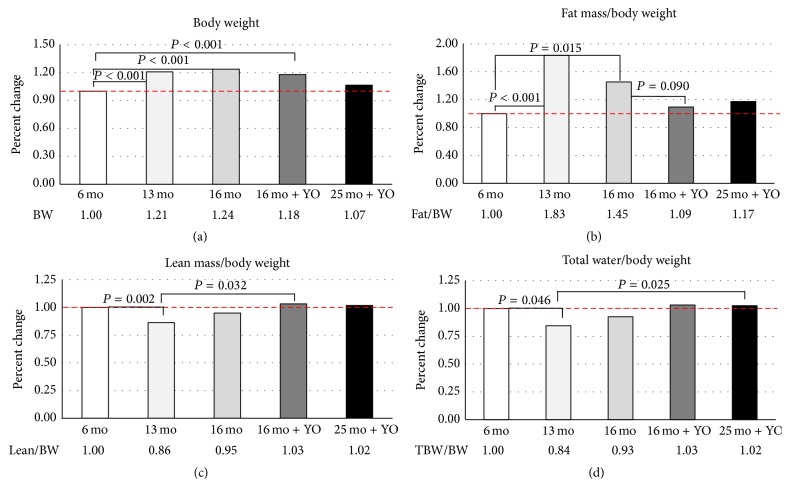
Body composition. MRI analysis detected significant age-associated changes in body composition. (a) Body weight was significantly increased with age. (b) Fat mass was significantly increased with age and tended to be decreased with ovarian transplantation. (c) Lean mass was decreased with age and increased with ovarian transplantation. (d) Total body water was decreased with age and increased by ovarian transplantation. Values are reported as a percentage of the values in six-month-old control mice.

There were significant differences in body weight between age groups, but not due to treatment (Figure 4). Fat mass was significantly increased at 13 and 16 months of age in control mice (*n* = 5 and *n* = 5, resp.). Fat mass was reduced by 25% by ovarian transplantation in 16-month-old recipient mice (*n* = 5). The percentages of lean body mass and total body water were significantly reduced in 13-month-old control mice. The percentage of lean body mass and total body water in 16- and 25-month-old recipient mice (*n* = 5) was restored to the levels found in six-month-old control mice (*n* = 10) by ovarian transplantation.

## 4. Discussion

Six-month-old female CBA/J mice are reproductively competent and have active ovaries that are reproductively cycling. By 12 months of age, ovaries in female CBA/J mice have become senescent and the mice have ceased reproductive cycling. In the current experiments, a group of 12-month-old mice received young ovaries from two-month-old donors. Therefore, the six-month-old control mice and 16-month-old recipient mice both possessed six-month-old ovaries and both benefitted from the effects of relatively young ovarian function. Because the 16-month-old cohorts of mice were collected prior to death, we were unable to collect life span data on these mice. However, previous studies using the same ovarian transplantation model in the same strain of female CBA/J mice resulted in a significantly increased life span in transplant recipients [1]. Therefore, the 16-month-old transplant recipients used in the current experiments would likely have enjoyed an extended life span, compared with nontransplanted mice.

Skeletal muscle function declines with age [15]. Because the decline in muscle function impacts quality of life, assessment of muscle function represents an appropriate indicator of health span. To assess skeletal muscle function, in the current experiments we chose to use an inverted cling grip test. Grip strength decreases with age in C57BL/6 mice and can be a good predictor of remaining life span [16, 17]. The inverted cling grip test has advantages over standard grip strength and measures overall strength and endurance, similar to a pull-up test in humans [18].

In our mice, a significant loss of grip strength occurred from six to 16 months of age. Ovarian transplantation moderately (16%) exacerbated this loss of grip strength in 16-month-old mice. This was unexpected as ovarian transplantation increased lean muscle mass by 8% in 16-month-old recipient females. Transplant recipients also weighed 5% less than 16-month-old control females. A decline in muscle mass (sarcopenia) is frequently observed in postmenopausal women [19]. Previous human studies have demonstrated a significant increase in lean body mass due to hormone replacement therapy (HRT [20]). Contradictory studies have reported a significant loss of lean body mass due to HRT [21]. These reports demonstrate that the results of hormonal supplementation to treat sarcopenia in humans are often contradictory.

Mice are inherently afraid of falling and motivation may have influenced the performance on this test. Studies in postmenopausal women found that mean anxiety/fear scores improved significantly with HRT [22, 23]. Old mice with new ovaries may have been less fearful of falling than old control mice. Alternatively, the surgical procedure itself may have negatively influenced the grip strength of the transplant recipient mice. However, these mice had four months to recover from surgery prior to being tested and, in previous work, mice that underwent a sham surgical procedure were no different from nonsurgical mice four months postoperatively with respect to any physiological parameter measured. In this previous work, body weight was initially reduced in sham operated mice, but two months postoperatively there was no longer any significant difference in body weight between sham operated and nonsurgical control mice [1, 2, 9]. Therefore, in the current report, the decreased body weight in recipient mice was more likely due to the presence of new ovaries and not due to surgical after-effects. It should be noted that interventions that extend life span, such as ovarian transplantation, may modulate muscle biology independently of its effect on aging.

Glucose intolerance increases with age. As a measure of glucose tolerance, we used a standard GTT assay. We used an IPGTT rather than an oral (PO) GTT as the PO test is associated with an increased level of stress on the tested subjects and the PO test is also associated with gastrointestinal variability in the timing and dose of glucose absorbed. Variability in the IPGTT is often attributed to the injection needle penetrating the intestines and glucose being variably deposited into the gastrointestinal tract rather than the intraperitoneal cavity. We utilized short-needled (6 mm) insulin syringes to minimize this possibility. Aged mice are often intolerant of insulin injections and thus our metabolic testing was limited to the glucose tolerance test in the current experiments.

The glucose AUC was not significantly influenced by age or ovarian transplantation. However, in 16-month-old controls, 80% of the mice displayed a monophasic glucose curve (*n* = 4), but in 16-month-old mice with young ovaries only 60% displayed a monophasic glucose curve (*n* = 3) and 40% displayed a biphasic glucose curve (*n* = 2). Mice in all groups that displayed a biphasic glucose curve (*n* = 4) displayed lower fasting glucose levels, significantly greater body weight, heavier hearts and kidneys, lighter spleens, greater lean body mass, and lower fat mass and displayed increased grip strength, compared to mice with a monophasic curve (*n* = 21). The biphasic shape has been associated with normal glucose tolerance in humans and those with a monophasic plasma glucose curve have been shown to be at an increased risk for insulin resistance [24, 25]. These observations suggest that mice that display a biphasic glucose curve also display many other positive health span attributes. However, a conflicting observation was that only 10% of the six-month-old mice (*n* = 1) which were assumed to be healthier displayed a biphasic glucose curve. With the small numbers associated with these particular observations, any conclusions from these assessments are tenuous at best.

Normal aging in humans and rodents is characterized by a reduction in skeletal muscle mass as well as increases in adipose tissue mass. In the current study, body weight increased significantly with age but was significantly reduced in 16-month-old mice by ovarian transplantation. This reproduction-dependent reduction in body weight was mainly due to a large decrease in body fat. Total lean body mass was relatively unchanged by ovarian transplantation. There was also a large decrease in free water due to ovarian transplantation, but not in total body water (which was increased). Generally, total body water decreases with age, with the highest levels present at birth and a precipitous drop thereafter until death [26]. Restoration of total body water by ovarian transplantation may suggest restoration of a physiologically younger state. These trends in body composition changes in 16-month-old transplant recipients were mirrored in 25-month-old transplant recipients, but with slightly less influence of the transplanted ovaries.

Ovaries in our 13-month-old mice became senescent at about 10–12 months of age. Therefore, these 13-month-old mice have only been without active ovaries for approximately 1-2 months. It normally takes about 100 days for animals to respond/adjust to major changes in ovarian function (critical period hypothesis). The “critical period” hypothesis suggests that there is an interval during perimenopause where hormone replacement therapy is effective, but efficacy decreases if therapy is initiated in the postmenopausal years [27, 28]. The “critical period” is hypothesized to result from the loss of ovarian hormone receptors, resulting from long-term hormone deprivation, such as at menopause [29]. Supporting this idea is the finding that in rats long-term ovarian hormone deprivation attenuated the ability of hormone replacement therapy to regulate levels of estrogen receptor-*α* [11]. In the brain, long-term estrogen deprivation leads to loss of estrogen receptors [30] and both young and old mice with senescent ovaries are less responsive to estrogen treatment [31]. Therefore, our 13-month-old mice were likely in a period of adjustment, being without ovarian hormones for the first time, but not having had enough time to develop adaptive mechanisms.

Overall, the restoration of health by transplantation of young ovaries appears to be relatively robust. A major limitation of this study was the modest number of animals available in each treatment group. This is not an unusual situation in aging studies. However, even with the small number of animals used, many tests displayed large differences between groups but often failed to reach statistical significance.

## 5. Conclusions

In summary, we have shown that sarcopenia, which is negatively influenced by aging in females, can be positively influenced by reestablishment of active ovarian function in aged mice. However, much controversy remains regarding how the ovary provides the ovarian-dependent health advantage enjoyed by young females and its role in the restoration of health in aged females. These findings provide strong incentive for further investigation of the positive influence of young ovaries on restoration of health in postreproductive females.
